# The Association of Early-Morning Eating Habits With High Nutrient Intake by Older Japanese Adults Living Alone

**DOI:** 10.1093/geroni/igaa057.779

**Published:** 2020-12-16

**Authors:** Yoko Matsuoka, Junhua Zhang, Weida Lyu, Kei Aoyagi, Mai Takase, Ryogo Ogino, Jun Goto

**Affiliations:** The University of Tokyo, Tokyo, Japan

## Abstract

The consumption of essential nutrition is fundamental to maintain the health of older adults. Conventional studies report that community dwelling older adults who live alone have the tendency to skip meals and result in low nutrition intake. However, the details regarding the dietary behavior and its association with the status of nutrition intake remain unclear. In this study, a cross-sectional analysis was conducted to explore the association between the time of meals and nutrition balance. In October 2019, questionnaire surveys were distributed to 184 participants of a lunch event dedicated to older adults living alone (Kashiwa, Japan). The time of meals, number of meals per day, consumption status of 10 food groups, and self-rated health were used for the analysis (N=165). As a result, older adults who usually ate their first meal between 5 to 8 A.M. ate three meals/day, while those who ate their first meal after 8 A.M. ate two meals/day. Those who ate their meal between 5 to 8 A.M., frequently consumed meat, fish and seafood, milk, and green and yellow vegetables compared to those who ate after 8 A.M. Self-rated health score was also high. The consumption of early-morning meals was associated with good dietary behaviors. The importance of eating breakfast has been emphasized, however, the time of the breakfast could vary among individuals. This study proposed the importance of considering the time of the meal. Development of intervention programs which encourage early-morning meal consumption might be helpful to form healthy dietary behaviors of older adults.

